# New perspectives: systems medicine in cardiovascular disease

**DOI:** 10.1186/s12918-018-0579-5

**Published:** 2018-04-25

**Authors:** Frank Kramer, Steffen Just, Tanja Zeller

**Affiliations:** 1 Department of Medical Statistics, University Medical Center Göttingen, Humboldtallee, 32 Göttingen, Germany; 2 Molecular Cardiology, Department of Medicine II, University of Ulm, Ulm, Germany; 3 Clinic for General and Interventional Cardiology, University Heart Center Hamburg, Martinistrasse 52, 20246 Hamburg, Germany; 4 German Center for Cardiovascular Research (DZHK e.V.), Partner Site Hamburg, Lübeck, Kiel, Hamburg, Germany

## Abstract

**Background:**

Cardiovascular diseases (CVD) represent one of the most important causes of morbidity and mortality worldwide. Innovative approaches to increase the understanding of the underpinnings of CVD promise to enhance CVD risk assessment and might pave the way to tailored therapies. Within the last years, systems medicine has emerged as a novel tool to study the genetic, molecular and physiological interactions.

**Conclusion:**

In this review, we provide an overview of the current molecular-experimental, epidemiological and bioinformatical tools applied in systems medicine in the cardiovascular field. We will discuss the status and challenges in implementing interdisciplinary systems medicine approaches in CVD.

## Background

Cardiovascular diseases (CVD) represent one of the most important causes of morbidity and mortality worldwide, with an increase in the global number of death from CVD by 12.5% in the past decade [1]. Consequently, CVD poses a major public health burden with high socioeconomic impact. Clinical risk factors, such as age, sex, hypertension, diabetes mellitus, hyperlipidemia, and family history, are still the predominant indicators for likelihood of developing coronary artery disease [2].

However, innovative approaches to increase the understanding of the multifactorial, complex underpinnings of CVD promises to enhance CVD risk assessment and might pave the way to tailored therapies.

Despite success of genome-wide association studies [3–6] and sequencing approaches [7], the underlying pathophysiological mechanisms of CVD remain - in part, to be determined.

So far, diseases such as CVD are typically defined by late-appearing disease manifestation, by the range of clinical pathophenotypes, however, this definition neglects the underlying molecular pathophysiological disease mechanisms [8]. A diseases is rarely a simple consequence of an abnormal single effector but, rather, is a reflection of pathobiological processes interacting in a complex network [8].

To provide a more comprehensive picture, the systematic integration of multidimensional datasets evolves as an emerging, so called systems medicine approach including molecular findings of regulatory RNAs and DNA, proteins, metabolites as well as knowledge from cell biology, animal experiments and human phenotypic and clinical data [9, 10].

**Fig. 1:**
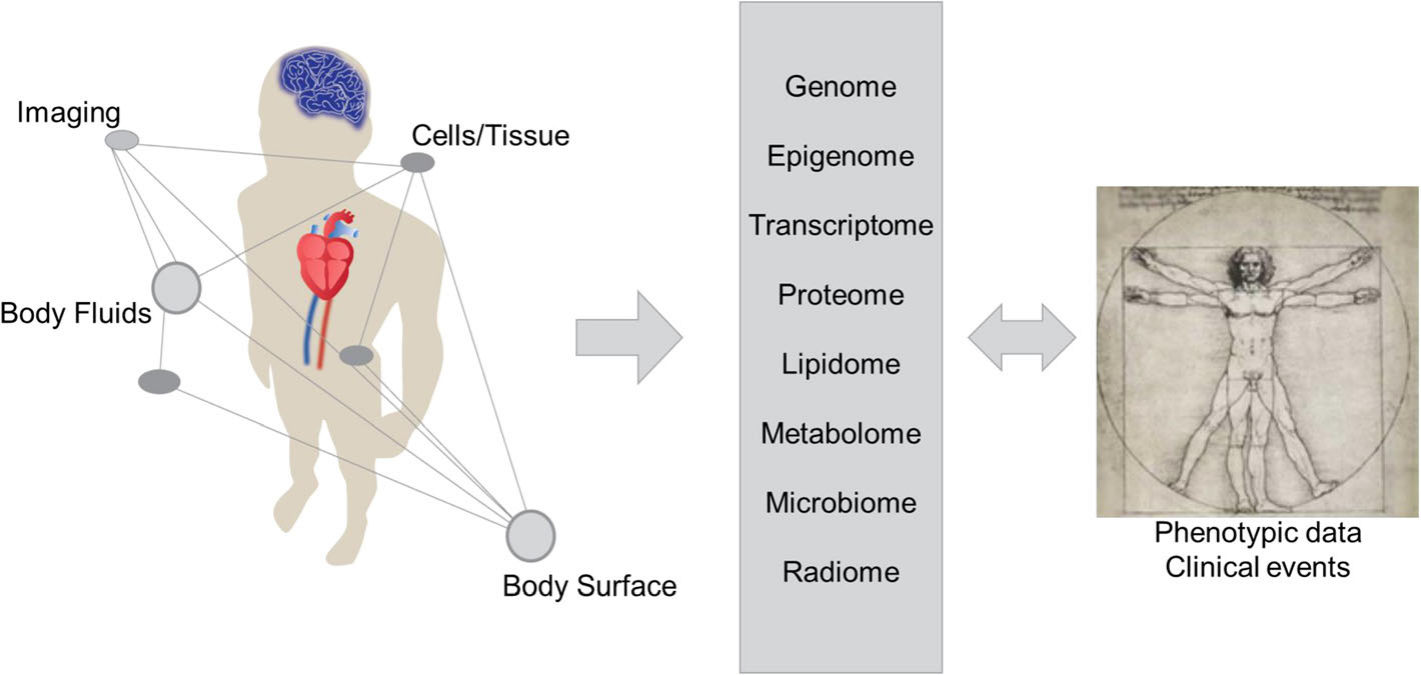
Overview of multidimensional omics and related clinical phenotypes used in systems medicine

One definition describes systems medicine as the implementation of systems biology approaches into medical research (https://www.casym.eu, [11]). This definition refers to research approaches intended to improve understanding of biological mechanisms through the use of omics-based science, systems biology, bioinformatics and network theory and shall promote the application of medical informatics tools to improve patient care [12–14]. This relatively new research field relies on interdisciplinary approaches involving clinicians, bioinformaticians and mathematicians, data management, engineers as well as epidemiologist and researchers in life science such as biologists and physicists. Therefore, collaborations across disciplinary boundaries and different “scientific languages” are crucial. Systems medicine makes use of the rapidly increasing amount of multidimensional omics and related medical and biological data spanning from clinical phenotypes and data from human studies to molecular experimental laboratory data [9] (Fig. 1).

**Fig. 2:**
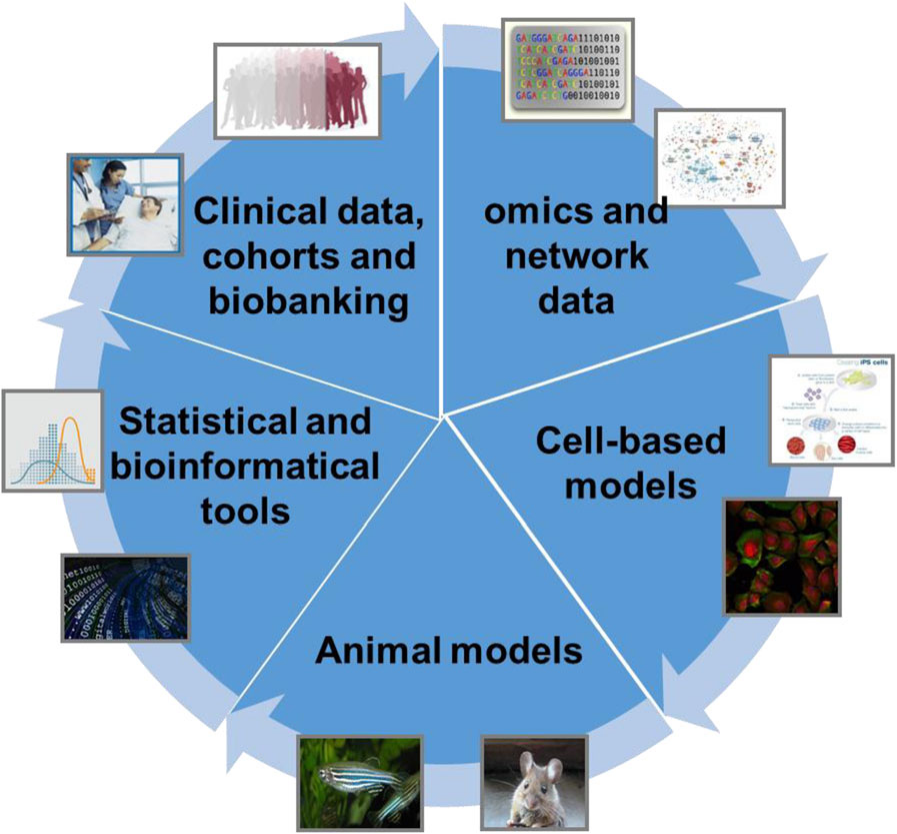
Overview about current molecular-experimental, epidemiological and analyses tools applied in systems medicine

In this review, we provide an overview of the current molecular-experimental, epidemiological and bioinformatical tools applied in systems medicine in the cardiovascular field (Fig. 2). We will discuss the status and challenges in implementing interdisciplinary systems medicine approaches in CVD.

## Description of current tools for systems medicine in cardiovascular disease

### Experimental tools

Decades of reductionist scientific approaches aimed on the elimination of complexity of the biological systems under investigation, thereby separately and successively defining the individual basic units of the entire system [15, 16]. In the context of human diseases, understanding complex and interconnected mechanisms merely by focusing on individual genes or signaling pathways is usually insufficient. With the entrance into the arena of high-throughput technologies and the capability of generation of massive amounts of multifaceted “omics data” from biological systems, systems biology has emerged as an interdisciplinary field of research that integrates the heterogeneous high-throughput data to manage this high complexity quantitatively and in a modeled fashion. Nevertheless, the prerequisite for the meaningful modeling is the use of the ideal model systems for “omics data generation”. In this regard, cell-based systems and animal disease models should accurately recapitulate the pathology observed in the patient.

#### Cell-based cardiac disease models

The vertebrate heart consists of different cell types such a cardiomyocytes, cardiac fibroblasts, endothelial cells or vascular smooth muscle cells that all significantly contribute to regular heart function [17]. Nevertheless, cardiac myocytes are the main cardiac cell population and predominately contribute to cardiac dysfunction in human patients and cardiac disease models. Isolated primary neonatal cardiomyocytes from mice and rats are excellent sources for the investigation of distinct gene functions, cellular processes and molecular alterations under physiological as well as pathophysiological conditions in vitro [9]. These cells are easy to isolate and to purify as well as to culture for up to 28 days. Additionally, these cultured cells beat spontaneously, are transfectable, and has been shown to be useful to study myofibrillogenesis, myofibrillar functions, hypertrophic responses and to model cardiac diseases [17]. In this context, the availability of numerous genetic mouse models of human cardiac disease allows the isolation of primary neonatal cardiomyocytes directly from these mouse models providing the opportunity to evaluate the functional, structural and molecular roles of these factors in cardiomyocytes in detail. Unfortunately, primary neonatal cardiomyocytes display a rather immature phenotype hindering the investigation of cellular and molecular processes solely present in adult/terminally differentiated cardiomyocytes. By contrast, adult mammalian primary cardiomyocytes are terminally differentiated allowing the complementary assessment of functional, structural and molecular gene functions in a mature cardiomyocyte context. Nevertheless, isolation of adult primary cardiomyocytes is technically challenging and isolated cells can only be cultured for a short period of time, fail to beat spontaneously and are extremely difficult to transfect, significantly impeding the analyses of specific gene functions and molecular mechanisms [17]. Furthermore, some effort has been put in the development of cardiac cell lines by using immortalized cardiomyocytes such as AT-1, HL-1, ANF-T-antigen and H9C2 cells. Whereas AT-1, HL-1 and ANF-T-antigen cells were derived from mouse atrial cardiomyocyte tumors, H9C2 cells were obtained from embryonic rat ventricular tissue [17]. Although these cardiomyocyte cell lines are valuable tools to answer defined scientific questions, unfortunately none of these cell lines recapitulates the exact physiological properties of primary cardiomyocytes therefore limiting their use in the in vitro assessment of precise and complex gene functions.

Embryonic stem cells (ESC) have been used for decades to generate cardiomyocytes and recent advances in cell culture techniques significantly increased the yield of cardiomyocytes in these cultures. Although ESC-derived cardiomyocytes can be rapidly expanded and easily manipulated, the cells are phenotypically immature and do not represent fully functional, terminally differentiated cardiomyocytes [17, 18]. Induced pluripotent stem cells (iPSCs) are reprogrammed cells that show similar features and properties than ESCs including the capability of differentiating into cardiomyocytes [17–19]. IPS cells can be generated directly from somatic cells by introducing a cocktail of transcription factors. One great advantage of iPS cells is that these cells can easily obtained from patients with a genetic disease thereby representing an invaluable tool for human disease modeling. Additionally, similar to ES cells, iPSC-derived cardiomyocytes are easy to manipulate and state-of-the-art differentiation protocols lead to high percentages of cardiomyocytes in these cultures. Remarkably, depending on the differentiation time, iPSC-derived cardiomyocytes are assumed to resemble an immature, embryonic phenotype (2–3 weeks after differentiation) or a more mature “fetal” phenotype (12–15 weeks after differentiation) allowing the investigation of both, developmental processes as well as functional, structural and molecular parameters solely present in more differentiated cardiomyocytes [17]. Due to these invaluable features iPSC-derived cardiomyocytes are currently the best in vitro model for human cardiomyocytes. Nevertheless, compared to human adult cardiomyocytes, human iPSC-derived cardiomyocytes are smaller, beat spontaneously (sign of immaturity), display less structured myofibrillar organization and lack t-tubules, just to mention a couple of differences and shortcomings. Interestingly, human iPS cells can be used to generate engineered heart tissues (EHTs)/human cardiac organoids (hCOs), three-dimensional, hydrogel-based muscle constructs [20–22]. EHTs start to beat 10 days after culturing allowing the measurement of several aspects of contractile function in vitro. Compared to 2D cardiomyocyte cultures, conditions in EHTs appear more physiological, stable and maturated, however, EHTs using the current culturing techniques do still not reach the maturation status of adult primary cardiomyocytes yet.

#### Animal models of heart disease

In addition to cell-based disease models, modern biomedical research requires adequate animal models to understand the pathogenesis, progression, and pathomechanisms underlying human cardiovascular diseases (CVD). The ideal animal model system should be of low-cost and uncomplicated care, housing and breeding, manipulations and genetic modifications should be easy and reproducible and most importantly physiology should be comparable to the human situation to be able to translate experimental findings. Although various animal models ranging from small to large animals are frequently used in cardiovascular research, in this review we will focus on small animals to model CVD.

In cardiovascular research, rodent models such as mice, rats, rabbits or guinea pigs are the most frequently used animal models [23]. Maintenance is easy and cost-effective, they have a short generation time and genetic manipulations are possible. Additionally, rodent models enable the standardization of important parameters such as genetic background, age, temperature, diet or environment that is fundamental for the effective assessment of the pathogenesis and pathomechanisms of complex human cardiovascular diseases. Among all rodent models, mice are most frequently used in cardiovascular research although the murine cardiovascular system and cardiac physiology significantly differs from the human system. Their hearts are relatively smaller, heart rate is much faster and blood volume is much lower [9]. Nevertheless, during the last 20 years, the targeted generation of generically modified mice via embryonic stem (ES) cell injection such as knock-out or knock-in mice as well as the generation of transgenic mice led to the establishment of numerous meaningful disease models that mimic at least some of the characteristics of human cardiovascular disease. Using these models, it became clear that very similar pathways and mechanisms regulate the development, function and pathogenesis of the murine and human cardiovascular system. Additionally in mice, a large toolbox for the invasive and noninvasive manipulation of the disease model exists [23]. For instance, modeling human myocardial infarction (MI) in adult mice by the ligation of the left anterior descending (LAD) artery and the investigation of the cellular and molecular processes after MI is a valuable tool to understand the mechanisms of cardiac remodeling and repair. Interestingly, in contrast to adult mice, MI modeling in neonatal mice by LAD ligation or myocardial cryoinjury revealed significant myocardial regeneration and functional recovery due to induced cardiomyocyte proliferation, introducing the neonatal mouse as a model to study the molecular and cellular processes of mammalian heart regeneration and the mechanisms that convert adult mammalian cardiomyocytes post-mitotic [24–26]. Furthermore, transverse aortic constriction (TAC) is an established method to develop pressure-overload-induced hypertrophy in mice and other small mammals. The disease condition develops from significantly increased blood pressure in the left ventricle and recapitulates similar syndromes in humans that develop in response to aortic stenosis or chronic hypertension. Interestingly, Doxorubicin (DOX)-treatment in mice causes clinical symptoms very similar to human heart failure accompanied by significantly reduced left ventricular (LV) fractional shortening and cardiac output [23]. This non-invasive method of heart failure induction is therefore a valuable tool to dissect the molecular and mechanistic underpinnings of human heart failure.

Over the past 25 years, non-mammalian vertebrates enter the arena of heart disease modeling. In particular, the zebrafish (*Danio rerio*) has emerged as a unique model system to study human cardiovascular disease [27–30]. As a vertebrate and in contrast to invertebrate models such as flies and worms, the zebrafish possesses a closed cardiovascular system and a multi-cambered heart. Interestingly, during the first 6–7 days post ferilization zebrafish development does not depend on a functional cardiovascular system since sufficient oxygen is delivered by passive diffusion from the surrounding water. Additional advantages of the zebrafish over mammalian models are the large number of progeny, its fast and extra-uterine development, its transparency during embryonic development and the ease of genetic manipulation. In this context, the unique combination of forward and reverse genetic approaches including genome-editing tools makes the zebrafish a powerful organism to generate and establish novel cardiovascular disease models [29]. Ethylnitrosurea (ENU)-mediated chemical as well as viral or transposon-driven insertional mutagenesis in zebrafish have been conducted since the late 1990’s and resulted in the identification of numerous mutant strains and the subsequent definition of novel disease genes and pathomechanisms [28, 31]. For instance, the zebrafish mutant line *main squeeze* was shown to carry a mutation in the Integrin-linked kinase (ILK) gene resulting in reduced PKB/Akt phosphorylation, defective cardiac stretch sensor function and thereby severe cardiomyopathy in zebrafish [32]. Interestingly, mutations in the human ILK gene are meanwhile described in patients suffering from dilated cardiomyopathy (DCM) [33], verifying the usefulness of forward genetic screens in zebrafish to define novel disease genes and mechanisms. In addition to these hypothesis-free approaches, reverse genetics techniques by targeted gene inactivation or overexpression (transient or transgenic) has led to the establishment of many more in vivo models to study the genetic and molecular underpinnings of cardiovascular diseases. Here, Morpholino-modified antisense oligonucleotides that interfere with regular protein translation or pre-mRNA splicing of the target mRNA or precision genome-editing tools such as the Clustered Regularly Interspaced Short Palindromic Repeats (CRISPR)/Cas9 system, TAL effector nucleases (TALENs) or Zinc-finger nucleases (ZFNs) are well-established methods in zebrafish to broaden our understanding of the physiological role of the individual gene and the associated molecular signaling cascades in vivo. Using a Morpholino-mediated reverse genetics approach, the sarcomeric Z-disk protein Nexilin was first described in zebrafish to cause contractile dysfunction and cardiomyopathy when inactivated [34]. Based on these findings, Nexilin mutations were also found in DCM patients, again demonstrating the feasibility of functional genomics approaches in zebrafish to identify and characterize novel genes and pathways controlling the development and function of the cardiovascular system. Interestingly and in contrast to adult mammals, zebrafish retain the ability to fully regenerate their hearts after injury throughout adulthood. After acute cardiac damage, spared cardiomyocytes de-differentiate, re-enter the cell-cycle, and proliferate to replace the damaged myocardial tissue [35, 36], facts that render the small animal model zebrafish an invaluable vertebrate system to decipher the genetic factors and molecular mechanisms controlling heart regeneration. Nevertheless, similar to rodent animal models, obvious and significant differences between zebrafish and humans exist particularly in regard to cardiovascular morphology and anatomy, physiology but also pathology. In this context, large animal models of cardiovascular disease such as dogs, pigs, sheep, goats or non-human primates display a considerably higher cardiovascular similarity to humans and thereby disease characteristics more similar to human CVD [37]. Unfortunately, in contrast to small animal models, husbandry of large animal models is expensive, more complex and bulky. Additionally, generation times are longer and litters usually smaller provoking significantly increased study times to reach adequate animal numbers and statistical power. Particularly, the use of dogs and non-human primates in basic biomedical research is increasingly declining due to ethical aspects, although their cardiovascular anatomy and physiology best resembles the human situation. The large animal models most often used in cardiovascular research are pigs since their cardiovascular system is very similar to humans in regard to blood composition, heart anatomy and size, lipid profiles and lipoprotein metabolism [9]. Additionally, pigs exhibit spontaneous atherosclerotic lesion development, demonstrating the usefulness of pigs in studying the cellular and molecular underpinnings of coronary artery disease [9, 37]. These cardiovascular similarities between large animal models and humans enable the effective translation of scientific findings, however, significant challenges still exist to the use of large animal models. Species-specific antibodies and bioassays are rare and only few genetically manipulated models such as transgenic or knock-out animals are available to the scientific community.

Very recently, fundamental technical innovations were made in terms of genome-editing approaches to generate genetically modified animal models. As already mentioned before, Zinc-finger nucleases (ZFNs), TAL effector nucleases (TALENs) and especially the Clustered Regularly Interspaced Short Palindromic Repeats (CRISPR)/Cas9 system now enables scientists to precisely modify the genomes of virtually all cells, animals and even humans [38, 39]. With these fundamental technical advancements tailored models, even large animal models, can be generated and established that will massively enhance the field of cardiovascular research. Especially, the combination of generation of relevant and tailored models of human cardiovascular disease and the use of state-of-the-art “omics” technologies followed by bioinformatic and systems biologic modeling will ultimately foster effective drug discovery and the development of targeted therapies for human cardiovascular diseases.

### Cohort studies and biobanking

Epidemiological cohort studies and human biobanks offer the basis to translate hypotheses or findings of experimental settings into humans. Cohort studies provide the opportunity to study cardiovascular phenotypes over time and a well-designed cohort study can provide powerful results [40]. Prospective studies are carried out from the present time into the future, whereas retrospective studies are carried out at the present time, but look to the past to examine disease events and outcomes [40]. One prominent example in the cardiovascular field is the Framingham Heart Study (FHS), which was initiated in 1948. The FHS is the longest running prospective cohort study and through > 65 years of discovery has contributed enormously to the understanding of various cardiovascular risk factors and to how these factors relate to the overall and cardiovascular-related mortality [41, 42]. Various other epidemiological cohort studies were implemented since then and have provided additional information on cardiovascular disease risk [43–47].

In the last decades, not only phenotypic data of the study participants have been collected, also biospecimens became an important resource in epidemiology. Nowadays, large biobanks are an integral part of a cohort’s infrastructure [48, 49] and build the basis for a large part of the biomedical research. The main biospecimens collected into these biobanks include blood samples, saliva, tonsil swaps, urine, feces, tear fluid, tissue samples and biopsies, as well as genetic material. These materials are suitable for modern molecular analysis and consequently are critical for translating advances in molecular biology and technologies into improved human health and provide new possibilities in the context of systems medicine [49].

### Public data sources, prior knowledge and data integration

Using existing and/or newly generated data and prior knowledge resources as a basis for further analysis is a fundamental aspect to incrementally increase the scope of systems medicine research and will be a major aspect for data integration challenges in the years to come. Many of the algorithms and approaches mentioned below have already been proposed and/or applied in the field of cardiovascular diseases e.g. [50–52].

Large consortia have collected and maintained cohorts of matching clinical patient, animal model and omics data (*TCGA* [53]*; GEO* [54]*,* and ArrayExpress [55]) and of cell line profiles (CCLE [56], and LINCS [57]). Available data include sequencing data for genomics and transcriptomics, microarray mRNA and miRNA data as well as mass spectrometry data for proteomic analyses as well as many other “omics” data types. Table 1 lists a number of well-known public omics data repositories.

**Table 1:** Overview about publicly available omic data resources

Name	Description	URL	Reference
1000 Genomesproject	The goal of the 1000 genomes project was to find most genetic variants with frequencies of at least 1% in the populations studied	http://www.1000genomes.org	
ArrayExpress	Archive of functional genomics data stores data from high-throughput functional genomics experiments	https://www.ebi.ac.uk/arrayexpress	
GEO Gene Expression Omnibus	Public functional genomics data repository supporting MIAME-compliant data submissions. Tools are provided to help users query and download experiments and curated gene expression profiles	http://www.ncbi.nlm.nih.gov/geo	
EBI Expression Atlas	Gene expression patterns under different conditions. Data sets are re-analyzed in-house to detect baseline and differential expression patterns	https://www.ebi.ac.uk/gxa	[103]
GXD The Mouse Gene Expression Database	Collection of gene expression data in rodents. Focus on development.	http://www.informatics.jax.org/expression.shtml	[104]
TCGA – The Cancer Genome Atlas	Aims to assess the value of large-scale multidimensional analysis of molecular characteristics in human cancer and to provide the data rapidly to the research community.	https://gdc.cancer.gov/	[53]
LINCS - The Library of Integrated Network-Based Cellular Signatures	Aims to create a network-based holistic understanding of biology by cataloging changes in gene expression and other cellular processes upon perturbation.	https://clue.io/	[57]
CCLE - The Cancer Cell Line Encyclopedia	A compilation of gene expression, chromosomal copy number and sequencing data from 947 human cancer cell lines with pharmacological profiles for 24 anticancer drugs across 479 of the cell lines.	http://www.broadinstitute.org/ccle	[56]
PRIDE – PRoteomics IDEntifications	Repository for proteomics data, protein and peptide identifications and post-translational modifications.	https://www.ebi.ac.uk/pride/archive	[105]
COPaKB	Proteome biology platform specifically for cardiovascular research	http://www.heartproteome.org	[106]

Vast amounts of biomedical knowledge are available from online databases. This ranges from genetic sequence information on GenBank [58] to protein information on UniProt [59] to various sites of knowledge on molecular interactions, many of which are collected on the Pathguide.org website [60], which currently lists over 600 resources related to biological pathway and interaction knowledge. The most prominent pathway databases are Reactome [61], the Network Data Exchange (NDEx) [62], the Kyoto Encyclopedia of Genes and Genomes (KEGG) [63], and WikiPathways [64] as well as disease-specific databases such as the Online Mendelian Inheritance in Man (OMIM) resource [65]. Databases focusing on molecular interactions include BioGRID [66], IntAct [67] and STRING [68]. Further knowledge sources include drug-target databases, which connect therapeutica and targeted proteins and disease-target databases, which contain diseases known to be associated with specific mutations. Table 2 lists a number of well-known databases containing knowledge about molecular interactions.

**Table 2:** Overview of databases containing biomedical knowledge

Name	Description	URL	Reference
Molecular Information			
GenBank	The NIH genetic sequence database, an annotated collection of all publicly available DNA sequences	http://www.ncbi.nlm.nih.gov/genbank/	[58]
UniProt	Provide a comprehensive and freely accessible resource of protein sequence and functional information.	http://www.uniprot.org/	[59]
Ensembl	A genome browser for vertebrate genomes that supports cross-species research in genomics, evolution, sequence variation and transcriptional regulation.	http://www.ensembl.org/	[107]
Signaling Pathways			
Reactome	A free, open-source, curated and peer reviewed pathway database.	http://reactome.org/	[61]
NDEx - the Network Data Exchange	An online commons to upload, share, and publicly distribute networks. Networks receive globally unique accession IDs and can be stored for private use, shared in pre-publication collaboration, or released for public access. Includes Pathway Interaction Database (NCI) and the Cancer Cell Maps Initiative databases.	http://www.ndexbio.org	[62]
WikiPathways	A database of biological pathways maintained by and for the scientific community using a wiki approach.	http://www.wikipathways.org	[64]
Metabolic Pathways			
MetaCyc	Database of non-redundant, experimentally elucidated metabolic pathways. It is curated from the scientific experimental literature and contains pathways involved in both primary and secondary metabolism, as well as associated compounds, enzymes, and genes	http://metacyc.org	[108]
KEGG - Kyoto Encyclopedia of Genes and Genomes	KEGG includes graphical diagrams and data of biochemical pathways including most of the known metabolic pathways and some of the known regulatory pathways.		[63]
Protein-Protein Interactions			
IntAct – molecularinteractiondatabase	Database system and analysis tools for molecular interaction data derived from literature curation or direct user submissions	http://www.ebi.ac.uk/intact	[67]
BioGRID – biological general repository for interaction datasets	Interaction repository with data compiled through comprehensive curation, containing protein and genetic interactions, chemical associations and posttranslational modifications	http://thebiogrid.org	[109]
STRING – protein–proteininteractionnetworks	Database of known and predicted protein–protein interactions, including direct (physical) and indirect (functional) associations	http://string-db.org	[68]
Other Interaction Knowledge			
DrugBank	Combines detailed drug data with comprehensive drug target and drug action information	http://www.drugbank.ca/	[110]
PharmGKB - Pharmacogenetics Knowledge Base	Contains genomic, phenotype and clinical information collected from ongoing pharmacogenetic studies	http://www.pharmgkb.org/	[111]
DiseaseConnect	Comprehensive knowledge base on mechanism-based disease connectivity	http://disease-connect.org/	[112]
Connectivity Map	A resource that uses transcriptional expression data to probe relationships between diseases, cell physiology, and therapeutics.	https://clue.io/	[113]
OMIM - Online MendelianInheritance in Man	A comprehensive knowledge base of human genes and genetic disorders compiled to support human genetics research and education.	http://www.ncbi.nlm.nih.gov/omim/	[114]

Many of these databases can be integrated into the R Framework for Statistical Computing. Examples for software packages which offer functionality to integrate database knowledge include the BioPAX-ontology [69], Systems Biology Markup Language (SBML) [70], the Human Proteome Organization (HUPO) Proteomics Standards Initiative standard for Molecular Interactions (HUPO PSI-MI) [71] and NDEx [72]. In combination with mapping services, for example BioMart [73], these packages enable the integration and merging of prior knowledge for further analyses. Table 3 includes a number of standards for encoding pathway knowledge and corresponding software for the integration of this knowledge.

**Table 3:** Overview of standards and tools for encoding and working with pathway knowledge

Name	Description	Reference	Software Tools
BioPax	A standard language to represent biological pathways at the molecular and cellular level and to facilitate the exchange of pathway data.	[69]	Biopax [115] Paxtools [116]
SBML - The Systems Biology Markup Language	An XML-based format for representing biochemical reaction networks. Software-independent language for describing common models, including cell signaling pathways, metabolic pathways, gene regulation, andothers.	[70]	libSBML [117]
SBGN - The Systems Biology Graphical Notation	A visual language to encode pathways in three different granularities: process diagram, entity relationship diagram and activity flow diagram.	[118]	LibSBGN [119]
HUPO-PSI Molecular Interaction format	A community standard data model for the representation and exchange of protein interaction data. This data model has been jointly developed by members of the Proteomics Standards Initiative (PSI), a work group of the Human Proteome Organization (HUPO).	[71]	PSICQUIC [120]
Cytoscape Cyberinfrastructure Network Interchange Format (CX)	A light weight REST-based aspect-oriented interchange protocol for generic network data exchange.	[121]	[122]

## Methods for integrated data analyses

A large variety of methods for system medicine analyses has been proposed. The aim of these methods is often to classify data in order to provide conclusions towards a diagnosis, treatment decisions, and patient prognosis or towards clinical research hypotheses. The majority of the methods in use are based on statistics and computer science. This includes regression models (e.g. linear or logistic regression), non-parametric statistics (e.g. Wilcoxon-rank-sum [74] and the Kolmogorov-Smirnov test [75] and time-to-event analyses (e.g. Cox Proportional Hazard Ratio [76] from the field of statistics. Examples for methods originating from the field of computer-science are clustering (e.g. [77] and machine learning algorithms (e.g. Support Vector Machines [78] and neural networks [79]. Finally, some methods are very much present in both fields, for example probabilistic graphical models (e.g. Bayesian networks [80] and Boolean networks [81].

While many of these basic methods for analyses and modeling can be applied to any kind of omics analyses, e.g. genomics, transcriptomics or proteomics, they are often mixed and extended in order to account for the heterogeneous data available in a clinical setting and to integrate underlying biomedical knowledge. Integrating multiple types of omics data, e.g. genome, epigenome, transcriptome, proteome or metabolome for combined analyses is often referred to as multi-level analysis. Furthermore, data of the same omics type across various diseases or species can be combined as well as combining these two approaches – leading to multi-level and cross-species or cross-disease integration and analysis. These kind of combined analyses promise further insights into a holistic understanding of the underlying disease mechanisms. Often, the aim of these more complex approaches is to enable a certain kind of validation of a hypothesis on another omics-level or to allow a generalization and specialization of prior findings in different diseases or model organisms.

A few of these approaches have already been proposed and/or applied in the field of cardiovascular diseases, for example by Huan and colleagues [50, 51], and Raffler et al. [52].

A multi-level integration analyses on high blood pressure (BP), one of the major cardiovascular risk factors, had been conducted by Huan et al. [50, 51]. To identify novel candidate genes involved in BP regulation, systems approaches were applied by computationally combining genetic, transcriptomic, and phenotype data. By this analysis, 34 distinct genes were identified in relation to BP, explaining 5–9% of BP variation. To further seek for molecular key drivers of BP regulation, co-expression networks were identified [50, 82], leading to different sub-networks which have been connected by the SH2B adaptor protein 3 (*SH2B3*). The role of *SH2B3* in the development of hypertension was investigated in Sh2b3−/− mice in response to low-dose angiotensin II supplementation [83]. In untreated Sh2b3−/− mice, kidneys and aortas of transgenic mice showed greater levels of inflammation, oxidative stress, and glomerular injury. After angiotensin II infusion these effects were accelerated. A strong indication that the predominant effect of SH2B3 on BP is mediated by hematopoietic cells, came from experiments of bone marrow transplantations of SH2b3−/− into wild-type which reproduced the hypertensive phenotype. A subsequent study identified the role of genes of the BP co-expression networks including CRIP1 and showed that CRIP1 gene expression was correlated to measures of cardiac hypertrophy and identified circulating CRIP1 protein levels as a potential biomarker for increased risk for incident stroke, a sequel of high BP [84].

## Challenges and limitations in systems medicine

**Fig. 3:**
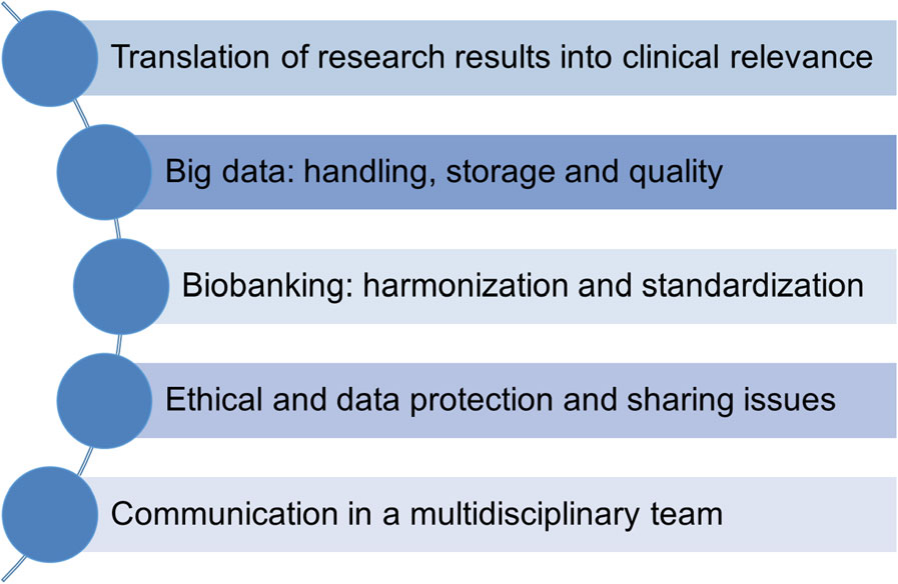
Challenges in systems medicine

The investigation of complex changes and interactions in the human body in an interdisciplinary team is one of the main advantages of systems medicine. In parallel, however, this is also one of the main challenges. Here, we provide an overview of the main challenges that needs to be considered (Fig. 3).

### The translation from animal models to human

Animal models are an important resource of cardiac research where a variety of cardiac processes and therapeutic targets can be studied. An ideal model system would be inexpensive, easily manipulated, reproducible, physiologically representative of human disease, and ethically sound [23].

Nevertheless, research findings in animal models are not always translatable into the human situation as most animal models – in particular small animal models, are somewhat artificial. Main reasons are that the metabolism of animals differs from that in humans, that the cardiovascular system of each animal has evolved differently in order to meet the demands of that species and that differences between the immune systems of animals and humans are potential confounders of animal experiments [85, 86]. Thus, depending on the cardiovascular process being studied, the choice of animal model needs to be considered carefully since it affects experimental outcomes and whether findings of the study can be reasonably translated to humans [85].

A first step to translate findings from small animal models is the use of experimental settings in larger species such as pigs or sheep. These large animal models can reflect the human situation more closely [85]. Nonetheless, the verification of findings in actual human samples and tissues is the most favorable way to shed further light onto translational applications for human patients [85] and one of the aims in systems medicine.

### Biobanks, big data management, quality and IT-infrastructure

Epidemiological cohort studies including comprehensive biobanks bear an enormous potential to study the underlying CVD pathophysiology and risk factors and thereby to successfully translate experimental or computational hypothesis into the clinical setting. In particular, the field of biobanking has increased in the last decades, and is going to be the largest ever library of biological materials [87].

Several challenges have evolved from the rapid rise of biobanks. One important issue is that, so far, no standardization of biospecimen handling, processing and storage practices is in place. Many different protocols and processes are used to collect, store, and analyze biological materials in biobanks. This poses a serious problem, as the quality of biospecimens and subsequently data quality is affected. The increasing size of collected biospecimens inevitably leads to large storage facilities including freezers and liquid nitrogen tanks, and additionally, to the need to replace manual protocols with automation. This large amount of biospecimens comes along with an incredible amount and complexity of data which need to be securely-stored and managed in data bases and subsequently, sufficiently quality-controlled.

Again, as the definition of system medicine states, handling and processing of biobanks cannot be achieved without interdisciplinary collaboration and networking [87].

With the advent of high-throughput technologies, life scientists need to cope with massive data sets, encountering challenges with handling, processing, harmonizing and moving/sharing information [88].

This challenge becomes even more prominent as omics and other data sets, which are not easily human-interpretable anymore, are increasingly generated in everyday healthcare. Successfully integrating these big data sources and developing an IT-infrastructure, which facilitates research as well as clinical routine, will be a major stepping stone for systems medicine. Clinical routine will require different levels of granularity of the information – ranging from a short overview of the patient record in the clinical information system up to an in-depth discussion of potential therapies at a tumor board review [89] or to inclusion for electronic decision support systems [90, 91]. Managing data and facilitating research will prove even more complicated because of the added complexity based on the integration of patient data, external omics data repositories, external knowledge sources and software frameworks for statistical analyses or machine learning approaches. Numerous approaches towards these challenges are in development, including the open electronics health records OpenEHR [91], the initiative “Informatics for Integrating Biology and the Bedside” (i2b2) [92–94] including the tranSMART software [94] and the cBioPortal for Cancer Genomics [95].

### Ethical considerations in systems medicine

Ethical considerations as well as issues of consenting patients for systems research are subject of current discussions worldwide. Because of the large number of participants in epidemiological studies and the long-term nature of these initiatives, traditional models of consent are considered impractical. Therefore, consent strategies have emerged that most often involve the use of some form of broad or open consent [96] to cover the multilevel assessment of personal, clinical and biological data as well as the storage and use of data in the biobanks [97].

Beside these considerations in terms of the use of data from subjects of epidemiological cohorts and experimental origin, further issues need to be discussed when medical informatics tools – e.g. electronic decision support and medical informatics systems, in the future shall be directly integrated and translated into clinical practice to support patients care. [12, 98–100]. For example, is it feasible that clinical decisions will be derived (only) from computer algorithms? What about concerns related to privacy, data protection and ownership of data?

### Multidisciplinary and communication

In the future, many researcher will be working as part of multidisciplinary teams [10]. This approach offers a wide range of perspectives and chances. Sharing expertise, knowledge, and skills will broaden the view of each team member and will have impact on their daily working. Addressing a cardiovascular clinical question on an interdisciplinary level will provide a more comprehensive picture of the disease status for both, researchers and clinicians and might guide decision-making processes in the future, as already performed in oncological tumor boards [101].

However, connecting experts in clinics, epidemiology, statistics, bioinformatics, and molecular- and cell-biology requires strategic efforts to motivate and sustain cross-disciplinary collaborations [11] and to advance a common “language” to better understand each other. A great and immediate challenge is to solve issues that arise from formal and legal requirements of data and biospecimen collection and sharing. These challenges include the development of new computational platforms for data integration, data handling and flow of information, the development of methodologies for statistical analyses of heterogeneous data sets and the improvement of concepts to integrate information across multiple levels (e.g. cell, tissue, organ, body) and multiple disciplines [11, 102]. Finally, data sharing activities also implicate ethical and legal issues related to data protection policies.

## Perspectives

For an efficient and successful implementation of systems medicine in the cardiovascular field important keys are: i) a sustained interplay and communication between experts of multiple disciplines, ii) optimized usage of resources and infrastructures to harmonize the environmental setting and to efficiently share data, biomaterial and knowledge, and iii) the extension of the research beyond traditional domains of discovery and disease etiology to accelerate translation into the clinics [10].

Major funding programs – publicly or private, need to support several disciplines at the same time, and need to invest in infrastructure. An integration of the industry into the systems medicine workflow can be advantageous for translating the knowledge into clinical trials. Additionally, researchers needs to acknowledge and foster ethical and data protection issues that inevitable evolve when working interdisciplinary and in the clinical field in system medicine.

Also from an economics perspective, it will be crucial to understand the costs and benefits of specific treatment options or diagnostic tests such as genetic testing [97] or by implementing electronic decision support.

What might be the next level of systems medicine in cardiovascular research?

In one scenario we will see a disease-spanning integration of data from the cardiovascular field with data from other chronic diseases to identify shared commonalities of risk factors across several forms of diseases [10].
